# Identification of *DNASE1L3* as a novel biomarker of clinical stage in liver hepatocellular carcinoma

**DOI:** 10.3389/fmolb.2025.1681888

**Published:** 2026-01-12

**Authors:** Weina Xue, Shuying Xie, Tong Wu, Ruixi Li, Dingyan Lu, Shuaishuai Chen, Yue Xu, Yonglin Wang

**Affiliations:** 1 State Key Laboratory of Discovery and Utilization of Functional Components in Traditional Chinese Medicine, Engineering Research Center for the Development and Application of Ethnic Medicine and TCM (Ministry of Education), Guizhou Key Laboratory of Modern Traditional Chinese Medicine Creation, Guizhou Medical University, Guiyang, China; 2 School of Pharmacy, Guizhou Medical University, Guiyang, China; 3 Leslie Dan Faculty of Pharmacy, University of Toronto, Toronto, ON, Canada

**Keywords:** bioinformatics, biomarker, clinical stage, *DNASE1L3*, liver hepatocellular carcinoma

## Abstract

**Background:**

Tumor staging is critical for guiding therapeutic decisions and determining prognosis in liver hepatocellular carcinoma (LIHC). This study aimed to identify potential tissue biomarkers intrinsically linked to disease stage to enhance our understanding of LIHC biology.

**Methods:**

Transcriptome and clinical data from LIHC patients were obtained from The Cancer Genome Atlas (TCGA) database. Differential expression analysis was conducted using the “limma” package. Weighted gene co-expression network analysis (WGCNA) was used to identify the gene module most strongly associated with LIHC and to extract hub genes. The hub genes then underwent differential expression, prognostic, and clinical staging analyses, immunohistochemical validation, and multivariable Cox regression analysis.

**Results:**

This analysis included data from 373 LIHC tumors and 50 solid tissue normal samples obtained from the TCGA database. Differential expression analysis identified 319 upregulated and 853 downregulated genes in LIHC tumors compared to these normal samples. An enrichment analysis highlighted key pathways, including cell cycle, DNA replication, and base excision repair. Three independent validation datasets confirmed 18 downregulated and 7 upregulated genes. Among them, *DNASE1L3*, *APOF*, and *FCN3* were consistently identified as core genes within the WGCNA-derived purple module. A further analysis using the UCSC database revealed that *DNASE1L3* and *APOF* were significantly associated with LIHC prognosis. A MEXPRESS analysis showed strong correlations between these genes and clinical stage, which was further supported by a SangerBox-based staging analysis, indicating significant differences in gene expression between early and advanced disease stages. Immunohistochemical data demonstrated that *DNASE1L3* levels decreased from stage I to stage III in LIHC. Multivariable Cox regression confirmed that low *DNASE1L3* expression is an independent predictor of poor prognosis in LIHC.

**Conclusion:**

Our results identified *DNASE1L3* as a promising tissue biomarker. Loss of *DNASE1L3* is indicative of advanced and aggressive LIHC, and therefore its expression may offer complementary information to current staging systems to improve prognostic assessment.

## 1 Introduction

Liver hepatocellular carcinoma (LIHC) is one of the most common malignant tumors worldwide and ranks third in tumor-related mortality (Chan et al., 2025). LIHC accounts for about 75%–80% of all liver cancer cases, rendering it a major global health problem (Sung et al., 2021). Unfortunately, LIHC often has no noticeable symptoms in its early stages, and therefore many patients are diagnosed at an advanced stage when treatment options are limited. Early detection and treatment are crucial for improving patient outcomes and reducing the burden LIHC places on affected individuals and healthcare systems (GBD 2015 Mortality and Causes of Death Collaborators, 2016).

LIHC has many causes, including viral infections, such as hepatitis B virus (HBV) and hepatitis C virus (HCV), and risk factors, such as obesity, diabetes, alcoholic liver cirrhosis, exposure to aflatoxin-producing *Aspergillus flavus*, and metabolic syndrome. The cause of LIHC varies significantly across different regions of the world. In the Asia-Pacific region and South Africa, LIHC is primarily caused by viral hepatitis, and particularly HBV (Sarin et al., 2020; Mak et al., 2018). In contrast, in the United States and Japan, HCV is the main cause of LIHC (Islami et al., 2017; Ko et al., 2021). Interestingly, the risk of HBV infection is higher during the perinatal period or early childhood (Higgins and O’Leary, 2023), although neonatal hepatitis B vaccination has been shown to effectively prevent mother-to-child transmission of HBV and reduce the incidence of LIHC due to chronic HBV infection (Wong et al., 2022). Chronic HCV infection can lead to metabolic disorders, steatosis, and liver cirrhosis, all of which are known risk factors for LIHC development (Khatun et al., 2021). LIHC has a high recurrence rate, with up to 70% of patients experiencing recurrence after treatment (Abdelhamed and El-Kassas, 2023).

A prospective study found that patients with diabetes are at a significantly increased risk of developing LIHC and cirrhosis (Pang et al., 2018). Moreover, alcohol-related liver disease and nonalcoholic fatty liver disease (NAFLD) are also significant risk factors for primary LIHC (Ueno et al., 2022; Xu et al., 2022). A cohort study found that the incidence of LIHC in patients with NAFLD was 17 times higher than that in the corresponding controls, a significant difference (Simon et al., 2021). Steatosis-related lipotoxicity and oxidative DNA damage were shown to increase the incidence of LIHC (Ioannou, 2021). Surgical resection and liver transplantation are effective early treatment methods for LIHC, with a 5-year survival rate of about 70%–80% (Ju and Yopp, 2020; Llovet et al., 2021). Other treatment methods include ablation, embolization, radiotherapy, chemotherapy, targeted drug therapy, and immunotherapy (Llovet et al., 2021). Although these treatments have demonstrated certain therapeutic efficacy, they still fail to significantly improve the quality of life or reduce mortality in LIHC patients. Thus, there is a critical need to discover reliable tissue-based biomarkers to guide accurate staging and personalized management throughout the LIHC disease continuum.

In this study, we performed a differential gene expression analysis on the LIHC transcriptome data obtained from The Cancer Genome Atlas (TCGA) database using the “limma” R package. We then subjected the differentially expressed genes (DEGs) to gene set enrichment analysis (GSEA) to investigate enrichment based on gene expression differences. Our analysis revealed that the cell cycle and DNA replication signaling pathways were significantly upregulated in LIHC patients, indicating dysregulation of the cell cycle in LIHC. Next, we used weighted gene co-expression network analysis (WGCNA) to determine that the purple module exhibited a strong correlation with the tumors (*r* = 0.80, *p* < 0.0001). This suggested that the purple module was highly correlated with tumor development in LIHC patients. Further screening of the purple module identified *DNASE1L3* and *APOF* as the genes most strongly correlated with the clinical stage of LIHC. Furthermore, immunohistochemical staining showed that DNASE1L3 was progressively lost in tumors in the progression from stage I to stage III. Multivariable Cox regression established low *DNASE1L3* expression as an independent prognostic factor in LIHC. Therefore, this study revealed that *DNASE1L3* expression is a tissue-based indicator of tumor stage and provides deeper insights into its role in LIHC prognosis.

## 2 Methods

### 2.1 Micro-matrix data information downloading and data processing

We obtained mRNA-Seq transcriptome quantification data and clinical information of LIHC patients from the TCGA website (https://www.cancer.gov/tcga). To standardize the genomic data, we normalized expression profiles using fragments per kilobase of transcript per million mapped reads (Song et al., 2017) and converted gene IDs using the SangerBox online resource (http://vip.sangerbox.com/home.html).

### 2.2 Data processing of differential gene expression

We performed a differential analysis of gene expression profiles between the LIHC and normal control groups using the “limma” R package, which uses generalized linear models to manage complex experimental designs and gene-to-sample variability (Ritchie et al., 2015). Prior to analysis, we excluded genes with an expression value of zero in more than 50% of the samples and transformed the data using the “voom” function. We used lmFit to perform a multiple linear regression analysis and then computed moderated *t*-statistics, moderated *F*-statistics, and log-odds of differential expression using an empirical Bayes moderation of standard errors toward a common value provided by the eBayes function. To identify statistically significant DEGs, we set a threshold of |log2FC| ≥ 2 and adjusted *p*-value <0.01.

### 2.3 Function and pathway enrichment analysis

To analyze the data, we used GSEA software (version 3.0) obtained from the GSEA website (http://software.broadinstitute.org/gsea/index.jsp). We divided the data into two groups based on clinical sample information and downloaded the Kyoto Encyclopedia of Genes and Genomes (KEGG) subset from the Molecular Signatures Database (http://www.gsea-msigdb.org/gsea/downloads.jsp) to evaluate pathways and molecular mechanisms related to LIHC (Liberzon et al., 2011). Using gene expression profiles and phenotype groupings, we defined the minimum gene set size as 5 and the maximum as 5,000, and performed 1,000 resamplings to enhance statistical accuracy. Significant enrichment was defined as a *p*-value of <0.05 and false discovery rate of <0.25 (Subramanian et al., 2005). We conducted Gene Ontology (GO) and KEGG enrichment analyses using the DEGs to investigate the underlying pathways involved in LIHC occurrence and development. Finally, we represented the results in the form of a chord diagram and bubble plot.

### 2.4 Construction and module division of the co-expression network

We used the “WGCNA” R package to construct a scale-free co-expression network based on LIHC patient transcriptome data. Our aim was to identify gene modules and hub genes that may be associated with LIHC development and progression. To generate the co-expression network, we first constructed Pearson’s correlation matrices and performed average linkage hierarchical clustering of gene groups with similar expression patterns to form modules. We set the minimum group size to 80 genes in the dendrogram and the sensitivity parameter to two. We then constructed a weighted adjacency matrix using a power function that emphasized strong correlations between genes and penalized weak correlations, with the soft-thresholding parameter β power chosen to achieve a scale-free topology. We then transformed the adjacency matrix into a topological overlap matrix (TOM). This measured the network connectivity of a gene as the sum of its adjacencies with all other genes, with the dissimilarity calculated as 1–TOM. To further analyze the gene modules, we calculated the dissimilarity of the module eigengenes and merged modules based on a distance threshold of less than 0.3. The grey module, representing a null gene set, was excluded from subsequent analyses. To identify hub genes, we calculated gene significance (GS) as a function of gene expression and specific traits and module membership (MM) by correlating gene expression with module eigenvectors. We applied a cutoff threshold of |MM| > 0.7 and |GS| > 0.7 to identify highly connected hub genes that may influence LIHC development (Tang et al., 2018).

### 2.5 Differential expression of core genes in normal and LIHC tissue

To compare differences in core gene expression between LIHC tumor tissue and normal tissue, we downloaded a standardized pan-carcinoma dataset (TCGA TARGET GTEx) from the UCSC Xena Browser (https://xenabrowser.net/). Using R software (version 3.6.4), we calculated the difference in gene expression between normal and LIHC tissue samples and conducted statistical testing using unpaired Wilcoxon rank-sum and signed-rank tests.

### 2.6 Differential expression of core genes in LIHC and normal tissue and the relationship with patient prognosis

To investigate whether differential expression of core genes between tumor and normal tissues affected the prognosis of patients with LIHC, we obtained a dataset from a previous TCGA prognostic study (Liu et al., 2018). We supplemented this dataset with TARGET follow-up data from the UCSC Xena Browser database and excluded patients with follow-up times of less than 30 days. To determine the optimal threshold value of the core genes, we used the “maxstat” R package (version 0.7–25), which applies a data-driven approach to identify cutoff values in the gene expression data. We set the minimum sample size threshold to <25% and the maximum sample size threshold to <75%. The identified optimal threshold value was used to divide the patients into groups with high or low gene expression. To investigate whether there were differences in prognosis between the high and low groups, we used the “survfit” function from the “survival” R package. This function estimates and visualizes a survival curve for each group and compares the curves using a log-rank test to assess significance. These analyses can help to identify potentially prognostic core genes in the core module and help clarify their role in LIHC progression.

### 2.7 Validation dataset: acquisition and analysis of gene expression Omnibus (GEO) datasets

To validate the differences in the expression of core genes between LIHC and normal tissue, we downloaded and analyzed three publicly available GEO datasets (accession numbers: GSE36376, GSE64041, and GSE112790). GSE36376 contained 433 samples (193 control and 240 LIHC samples), GSE64041 contained 120 samples (60 control and 60 LIHC samples), and GSE112790 contained 198 samples (15 control and 183 LIHC samples). We then used the GEO2R tool (https://www.ncbi.nlm.nih.gov/geo/) with the “limma” package to analyze DEGs in the LIHC tumor tissue compared to normal tissue, and visualized the results as a volcano plot. To identify DEGs common to all three GEO datasets, we used a Venn diagram. In addition, we used the GEPIA (http://gepia.cancer-pku.cn/) database to investigate the differential expression of the core genes between LIHC and normal tissue.

### 2.8 Clinical classification and core gene expression analysis

To investigate the potential association between the core genes in the core module and the clinical stage of LIHC, we used the MEXPRESS database (https://mexpress.be/) to query the correlation between the core genes and clinical indicators. Additionally, we downloaded the standardized TCGA pan-cancer dataset from the UCSC database for analysis. We extracted expression data for *DNASE1L3* and *APOF* genes of each sample and selected only LIHC samples.

### 2.9 Tissue microarray and immunohistochemistry (IHC) staining

A commercially available human LIHC tissue microarray (D106Lv01), which contained 29 stage I, 31 stage II, 27 stage III, and 12 stage IV LIHC tissue samples, was purchased from Xi’an Zhongke Guanghua Bioaitech Co., Ltd. Rabbit anti-APOF (16608-1-AP, Proteintech) and mouse anti-DNASE1L3 (67041-1-Ig, Proteintech) were used for IHC.

After deparaffinization in xylene substitute and rehydration through three changes of absolute ethanol, antigen retrieval was conducted in Tris-EDTA buffer (pH 9.0) using a microwave oven. Endogenous peroxidase was quenched with 3% H_2_O_2_ (25 min, room temperature, in the dark). After blocking with 3% bovine serum albumin (30 min), the sections were incubated with the primary antibody against DNASE1L3 (1:200) overnight at 4 °C. Detection was achieved with an HRP-polymer-conjugated goat anti-mouse IgG secondary antibody (1:200; GB23301, Servicebio) followed by DAB development. The staining for APOF was performed analogously, using its respective primary antibody (1:100) and an HRP-conjugated goat anti-rabbit IgG (1:200; GB23303, Servicebio).

IHC staining for DNASE1L3 and APOF was quantified with the Aipathwell digital-pathology platform (Servicebio). The deep-learning algorithms were used to identify tissue regions, classify positive staining by hue-saturation-intensity color models, and output staining parameters. Protein expression is reported as the histochemistry score (H-score): (1 × % weak) + (2 × % moderate) + (3 × % strong), giving a 0–300 scale in which higher values indicate greater expression.

### 2.10 Multivariable Cox regression

To identify independent prognostic factors, we performed multivariable Cox proportional hazards regression analysis using the “survival” R package. The model incorporated key clinical variables: age, sex, clinical stage, and *DNASE1L3* expression status. *DNASE1L3* expression was dichotomized into high and low groups based on an optimal cut-off value determined with the “survminer” R package. Results are expressed as hazard ratios (HR) with 95% confidence intervals (CI) and are visually summarized in a forest plot.

### 2.11 Statistical analyses

Error bars in the graphs indicate mean ± standard deviation (s.d.). Statistical significance was assessed using one-way analysis of variance in GraphPad Prism (version 9.0.0; GraphPad Software). A *p*-value <0.05 was considered statistically significant.

## 3 Results

### 3.1 Bioinformatics analysis results of the TCGA LIHC patients

**FIGURE 1:**
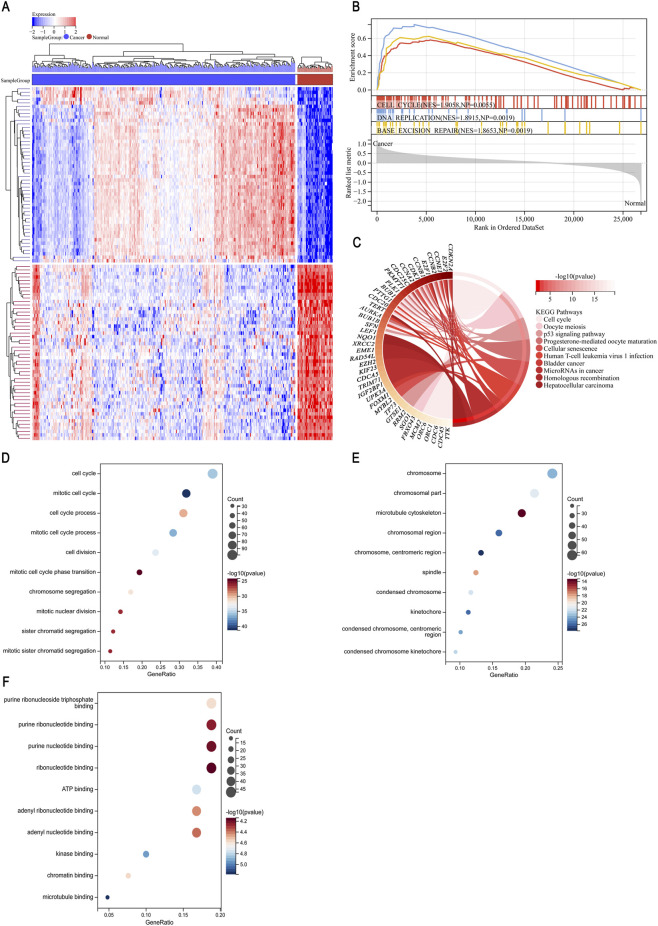
TCGA LIHC bioinformatics analysis results. **(A)** Heat map of the hierarchical cluster analysis of LIHC gene expression. **(B)** Enrichment plot of GSEA (top three): cell cycle, DNA replication, and base excision repair. **(C)** KEGG enrichment analysis map of upregulated DEGs. **(D–F)** GO (biological process, cell component, and molecular function) enrichment analysis map of upregulated DEGs.

We included a total of 373 LIHC tumors and 50 solid tissue normal samples in subsequent analyses. Hierarchical clustering of all the tissue data is shown in Figure 1A, in which darker colors indicate greater differences in expression. According to the results of the Limma analysis, LIHC patient tissue samples had 319 significantly upregulated genes and 853 significantly downregulated genes compared to normal tissue samples. We imported the expression profiles of the DEGs obtained by the Limma analysis into the GSEA analysis tool and identified the upregulated and downregulated enriched signaling pathways. Table 1 shows the top five GSEA pathway enrichment results, and Supplementary Table S1 shows the remaining results. In the results shown in Figure 1B (with only the top three results included), the top part of the picture indicates the enrichment fraction. The red curve (normalized enrichment score (NES) = 1.9058, nominal *p*-value (NP) = 0.0055) represents the cell cycle signaling pathway, the blue curve (NES = 1.8915, NP = 0.0019) represents the DNA replication signaling pathway, and the yellow curve (NES = 1.8653, NP = 0.0019) represents the base excision repair signaling pathway. The NES values for all three curves were greater than 1.8, indicating that these three pathways were significantly upregulated in LIHC. We imported the upregulated DEGs into KEGG (Figure 1C) and GO (Figures 1D–F) for enrichment analysis. The cell cycle signaling pathway was significantly upregulated, which was consistent with the GSEA analysis results. The main biological process was the cell cycle, the main cell component was chromosomes, and the main molecular function was purine ribonucleoside triphosphate binding. These results suggested that LIHC may reduce the time threshold required for cell division and shorten the cell cycle of LIHC cells, promoting proliferation and thereby leading to the occurrence of LIHC.

**TABLE 1:** Enrichment results of GSEA pathways.

Term	ES	NES	*p*-value	FDR	FWER
Cell cycle	0.5824	1.9058	0.0055	0.1697	0.127
DNA replication	0.7607	1.8915	0.0019	0.1023	0.147
Base excision repair	0.6240	1.8653	0.0019	0.0964	0.182
Pyrimidine metabolism	0.4712	1.8400	0	0.0982	0.231
Homologous recombination	0.6562	1.8158	0.0076	0.1006	0.286
Drug metabolism cytochrome p450	−0.6051	−2.0217	0	0.0156	0.047
Nicotinate and nicotinamide metabolism	−0.6404	−2.0503	0	0.0132	0.034
Arachidonic acid metabolism	−0.5528	−2.1164	0	0.0091	0.016
Retinol metabolism	−0.6967	−2.1642	0	0.0063	0.008
Tryptophan metabolism	−0.7678	−2.1924	0	0.0095	0.005

### 3.2 Results of weighted gene co-expression network analysis

**FIGURE 2:**
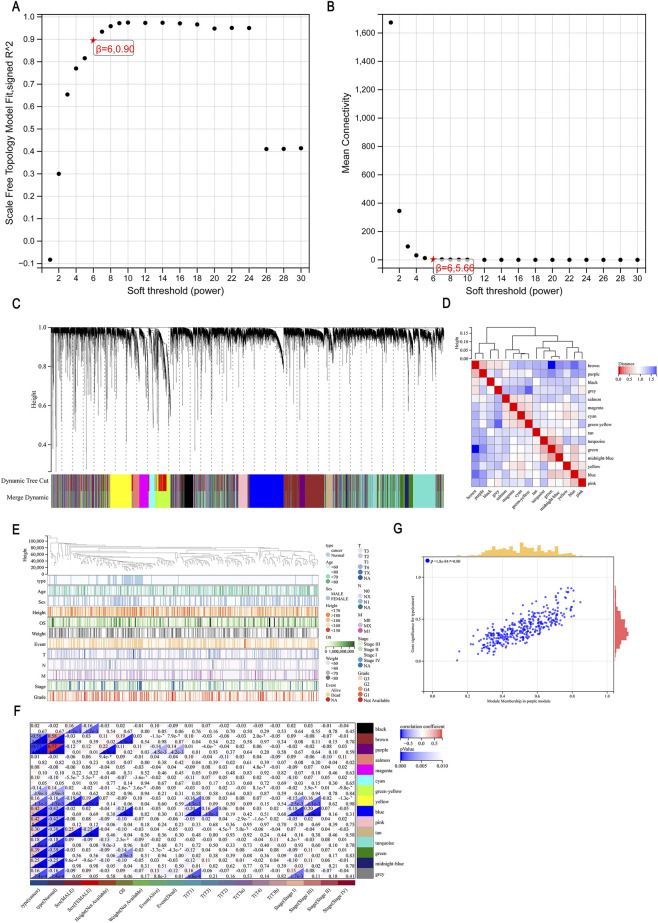
Clinical phenotype and module analysis of the WGCNA results. **(A,B)** Network topology analysis of various soft-thresholding powers. **(C)** Clustering dendrogram of genes, with dissimilarity based on the optimal topological soft threshold, together with assigned module colors. **(D)** Eigengene adjacency heatmap. **(E)** Distribution map of clinical trait clusters. **(F)** Module–feature association heatmap. **(G)** MM versus GS of the purple module.

To better conform the constructed network to a scale-free topology, we optimized the soft threshold, as shown in Figure 2A (scale-free fitting index, y-axis). The red asterisk indicates a subjectively selected scale-free fitting index value of 0.90. Figure 2B shows the network connectivity using different soft thresholds. When the scale-free fitting index was 5.66, the minimum soft threshold producing a scale-free network was six, which we selected as the optimal soft threshold for subsequent analysis. We then constructed a co-expression network based on this optimal soft threshold to categorize genes into different modules. Figure 2C displays the 15 main module clusters: black, blue, brown, cyan, green, green-yellow, grey, magenta, midnight-blue, pink, purple, salmon, tan, turquoise, and yellow. Darker colors indicated a higher correlation with the modules. As seen in Figure 2D, the brown and purple, turquoise and green, and midnight-blue and green modules showed strong correlations. Figure 2E presents the cluster diagram of clinical phenotypes. We next computed the correlations and significance between modules and clinical features and generated a heatmap of the correlations. As shown in Figure 2F, deeper colors signified higher correlations, with red indicating a positive correlation and blue indicating a negative correlation. The purple module showed the strongest correlation with tumors. To determine the correlation between the MM and GS, we drew a scatter plot presenting the correlation analysis. The results suggested that genes in the purple module were highly correlated with tumors (*r* = 0.80, *p* < 0.0001, Figure 2G). We subsequently performed hub gene extraction on the purple module, which identified 15 genes, as shown in Table 2.

**TABLE 2:** WGCNA purple module hub gene extraction results.

Gene	Module	MM_R	MM_*p*-value	GS_R	GS_*p-*value
*ADAMTS13*	Purple	0.735174898735093	4.33448801622503e–73	−0.863221853	4.73455254070051e–127
*ANGPTL6*	Purple	0.775312888880498	4.77480831427025e–86	−0.872889579	2.76826594417333e–133
*APOF*	Purple	0.708779014847601	8.65511388428502e–66	−0.727914891	5.3748124179613e–71
*CFP*	Purple	0.718591250371168	2.09651727087195e–68	−0.812305557	1.25467463148337e–100
*COLEC10*	Purple	0.807268028741152	1.85350437281764e–98	−0.841391822	1.39936526899686e–114
*CRHBP*	Purple	0.767133307357209	3.39037734107885e-83	−0.826802177	2.95741399185179e–107
*CSRNP1*	Purple	0.846768642146563	1.80668286524094e–117	−0.702977346	2.71161375805621e–64
*DNASE1L3*	Purple	0.759359963863238	1.35971476922448e–80	−0.761496657	2.67819579493704e–81
*ECM1*	Purple	0.777076021639937	1.11821243639271e–86	−0.825449758	1.30313123769237e–106
*FCN2*	Purple	0.795239244694728	1.57991939708967e–93	−0.868367258	2.626717060166e–130
*FCN3*	Purple	0.706351644230636	3.69509803359367e–65	−0.777008757	1.182174255898e–86
*LIFR*	Purple	0.763465304433654	5.90368689177615e–82	−0.752764156	1.84438189708973e–78
*MARCO*	Purple	0.705787082829552	5.16784449268037e–65	−0.762348379	1.39476642335384e–81
*OIT3*	Purple	0.774561624368027	8.82751310843317e–86	−0.820499579	2.66602999387227e–104
*PLSCR4*	Purple	0.808718025648439	4.46844738813903e–99	−0.713730096	4.27747293056936e–67

### 3.3 Relationship between core gene expression and prognosis

**FIGURE 3:**
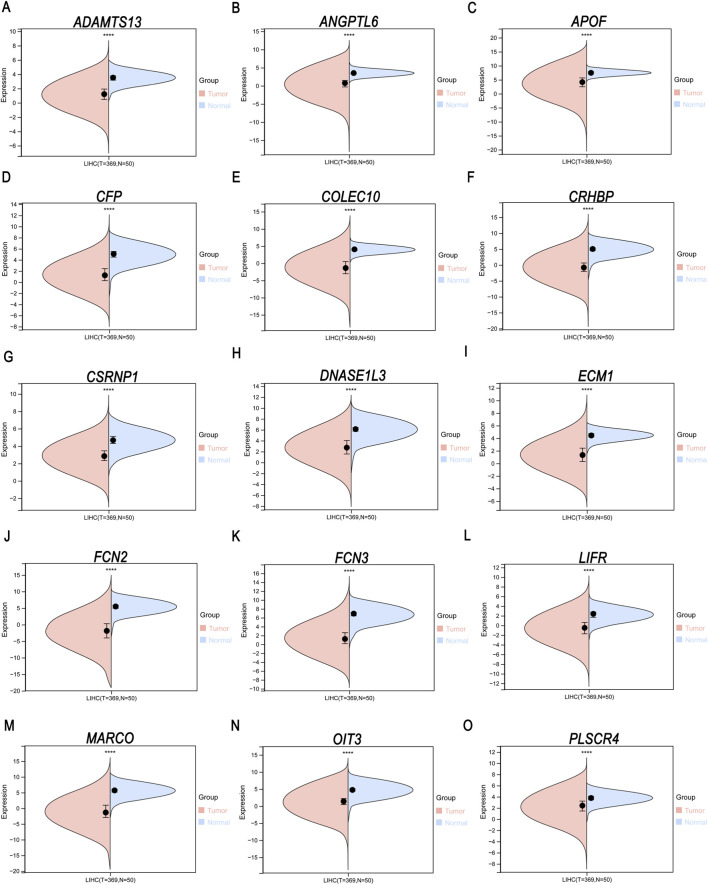
**(A–O)** Differential expression analysis of 15 core genes (*ADAMTS13*, *ANGPTL6*, *APOF*, *CFP*, *COLEC10*, *CRHBP*, *CSRNP1*, *DNASE1L3*, *ECM1*, *FCN2*, *FCN3*, *LIFR*, *MARCO, OIT3*, and *PLSCR4*) between normal and LIHC samples. ^****^ *p* < 0.0001.

We analyzed the gene expression of 50 normal tissue samples and 369 tumor tissue samples from LIHC patients using R software. As shown in Figure 3, the expression of all 15 genes was markedly lower in the tumor tissue samples than in the normal tissue samples (*p* < 0.0001). These findings suggested that these genes may affect the pathogenesis of LIHC.

**FIGURE 4:**
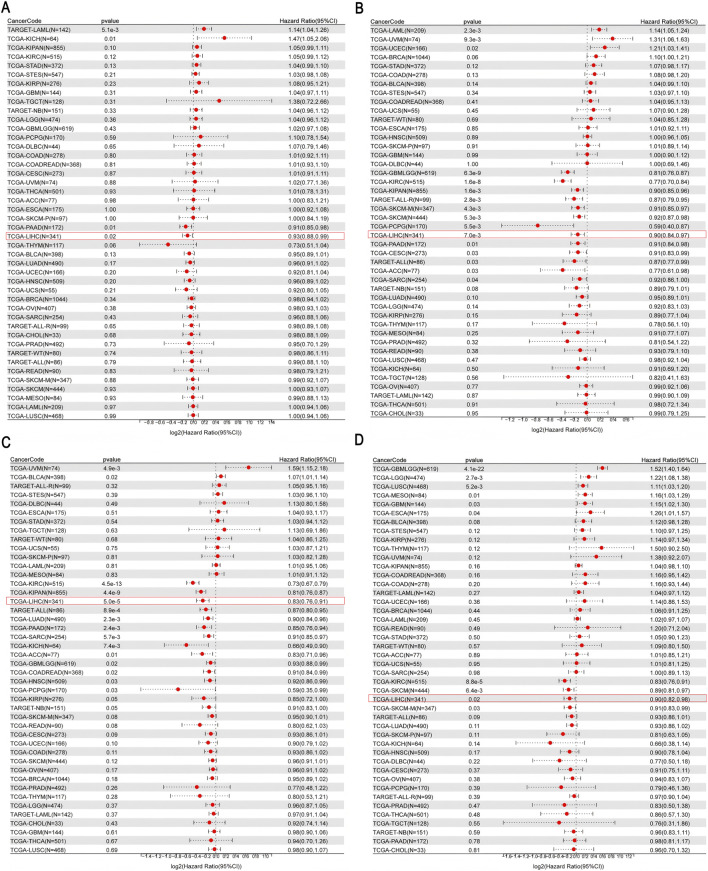
**(A–D)** Relationship between gene expression (*APOF*, *CRHBP*, *DNASE1L3*, and *FCN3*) and prognosis of LIHC patients.

To further elucidate the relationship between core gene expression and the prognosis of patients with LIHC, we analyzed 15 core genes using the UCSC standardized pan-cancer analysis tool. As shown in Figure 4, four genes, including *APOF* (*p* = 0.02), *CRHBP* (*p* = 7.0e–3), *DNASE1L3* (*p* = 5.0e–05), and *FCN3* (*p* = 0.02), were significantly correlated with the prognosis of LIHC and functioned as protective factors (risk ratio <1). Other genes were minimally correlated with LIHC patient prognosis (*p* > 0.05, Supplementary Figures S1–S3).

**FIGURE 5:**
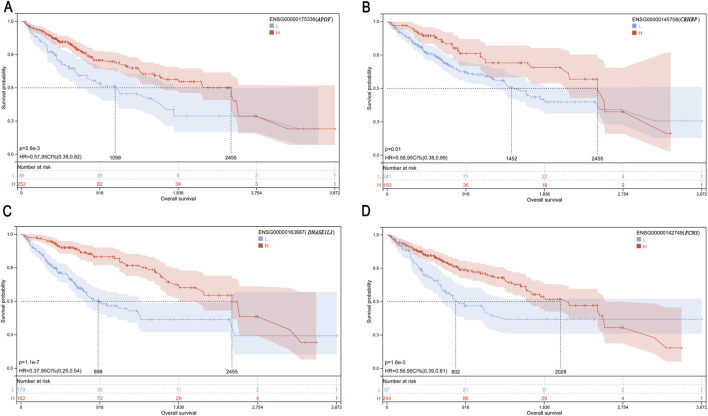
**(A–D)** Kaplan–Meier curves depicting patient survival based on gene expression (*APOF*, *CHRBP*, *DNASE1L3*, and *FCN3*).

We generated Kaplan–Meier curves using patient survival data based on the 15 core genes in LIHC (Figure 5; Supplementary Figures S4,S5). Our analysis revealed that patients with high expression of four specific genes, *APOF*, *CRHBP*, *DNASE1L3*, and *FCN3*, had a significantly better prognosis than those with low expression levels (*p* < 0.05, Figures 5A–D; Table 3).

**TABLE 3:** The optimal threshold value of the Kaplan–Meier curve.

Gene	Ensemble ID	Optimal truncation value	*p-*value
*APOF*	ENSG00000175336	2.6624	2.6e–3
*CRHBP*	ENSG00000145708	0.2400	0.01
*DNASE1L3*	ENSG00000163687	2.8381	1.1e–7
*FCN3*	ENSG00000142748	0.2029	1.6e–3

### 3.4 Volcano map of differential gene expression in the GEO dataset

**FIGURE 6:**
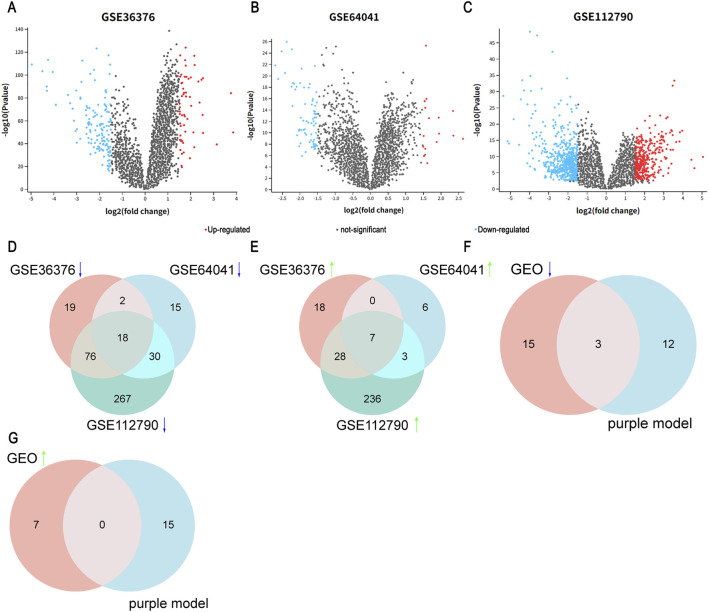
Volcano map of differential gene expression of the GEO datasets. **(A–C)** Differential gene expression of GSE36376, GSE64041, and GSE112790. **(D–G)** Interactive gene analysis between the GEO datasets (GSE36376, GSE64041, and GSE112790) and the purple module.

We applied the Limma analysis tool to the GEO datasets GSE36376, GSE64041, and GSE112790 as validation sets to obtain differential gene expression data, thereby further verifying the differential expression of core genes in LIHC tissue compared to normal tissue. As shown in Figure 6A (GSE36376), we identified 137 downregulated genes and 57 upregulated genes. As shown in Figure 6B (GSE64041), there were 71 downregulated genes and 17 upregulated genes, and as shown in Figure 6C (GSE112790), there were 589 downregulated genes and 343 upregulated genes. We also annotated the potential downregulated and upregulated genes. In GSE36376, there were 53 upregulated DEGs and 115 downregulated DEGs. In GSE64041, there were 16 upregulated DEGs and 65 downregulated DEGs. In GSE112790, there were 274 upregulated DEGs and 391 downregulated DEGs.

The diagram revealed 18 common downregulated genes (Figure 6D): *SDS*, *PGLYRP2*, *SLC22A1*, *SLCO1B3*, *LY6E*, *CYP1A2*, *FCN3*, *GBA3*, *GHR*, *CLEC1B*, *CLRN3*, *SHBG*, *HAMP*, *DNASE1L3*, *DCN*, *AKR1D1*, *APOF*, and *C9*. Additionally, seven common upregulated genes were identified (Figure 6E): *SPINK1*, *CAP2*, *ASPM*, *AKR1B10*, *GPC3*, *CCNB2*, and *TOP2A*. Furthermore, we intersected the common DEGs from the GEO datasets (GSE36376, GSE64041, and GSE112790) with the core genes of the WGCNA module to identify the genes in LIHC tissue. The results showed that three downregulated genes, *DNASE1L3*, *APOF*, and *FCN3*, were common to both the DEGs and the WGCNA module core genes, but no upregulated genes were common to both (Figures 6F,G).

**FIGURE 7:**
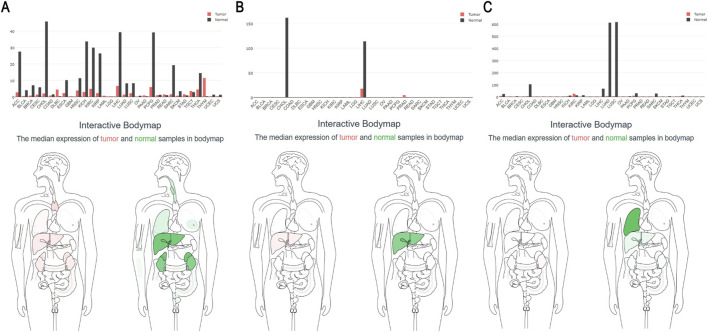
**(A–C)** Differential expression of *DNASE1L3*, *APOF*, and *FCN3* in tumor tissue compared to normal tissue using the GEPIA database data.

Based on the results from analyzing common genes from the GEO datasets and the purple module in the GEPIA database (Figures 7A–C), *DNASE1L3*, *APOF*, and *FCN3* were identified as protective genes with higher expression in normal tissue than in tumor tissue, with specific expression patterns across different types of tissue and tumors. *DNASE1L3* exhibited high expression across various types of tissue but low expression in several tumors; *APOF* was highly expressed in the liver and gallbladder but significantly decreased in patients with LIHC; and *FCN3* was mainly expressed in lung tissue. Based on this analysis, *DNASE1L3* and *APOF* were selected for further investigation of their clinical relevance.

### 3.5 Expression of *DNASE1L3* can predict the clinical stage of LIHC

**FIGURE 8:**
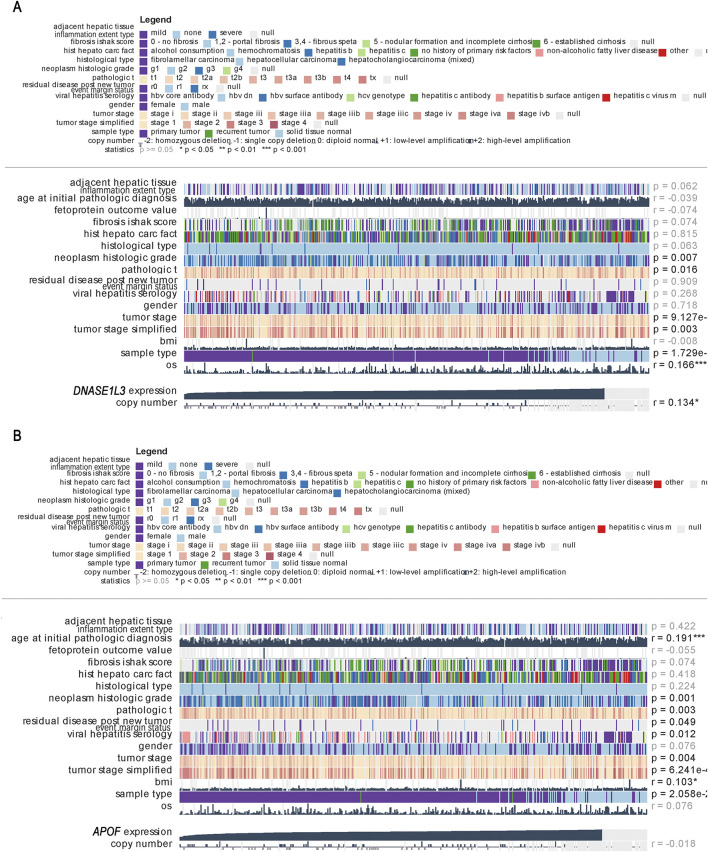
**(A,B)** Correlation between *DNASE1L3* and *APOF* expression and clinical indicators in the MEXPRESS database.

Tumor staging has significant clinical importance. Accurate tumor staging effectively guides patient treatment and provides important prognostic information and 5-year survival rates. We analyzed gene expression and clinical correlations. As shown in Figure 8, the results indicated that *DNASE1L3* and *APOF* expression significantly correlated with various clinical indicators, such as neoplasm histologic grade, tumor stage, and sample type. Based on these findings, we further investigated the association between *DNASE1L3* and *APOF* expression and tumor grading.

**FIGURE 9:**
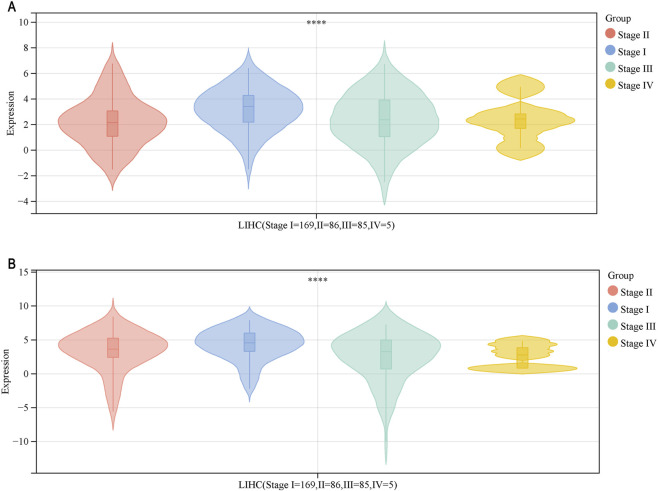
Gene expression and clinical typing of tumors. **(A)** Relationship between *DNASE1L3* expression and clinical stage. **(B)** Relationship between *APOF* expression and clinical stage. ^****^ *p* < 0.0001.

We analyzed *DNASE1L3* and *APOF* using the SangerBox gene expression and clinical typing module and found that both genes exhibited significant differences across different stages of LIHC (Figures 9A,B). As shown in Table 4, compared to stage I, *DNASE1L3* was significantly downregulated in stages II and III (*p* < 0.01). Similarly, *APOF* was significantly downregulated in stages II and III compared to stage I (*p* < 0.01), while differences in expression in other stages were not statistically significant.

**TABLE 4:** Statistical analysis between gene expression and the clinical classification of LIHC.

Gene	Number of patients	Comparable group (mean ± s.d.)	Control groups (mean ± s.d.)	*p*-value
*DNASE1L3*	LIHC (stage I = 169, II = 86, III = 85, IV = 5)	I (3.18 ± 1.59)	II (2.20 ± 1.85)	4.80e–05
		I (3.18 ± 1.59)	III (2.41 ± 1.97)	2.10e–03
		I (3.18 ± 1.59)	IV (2.42 ± 1.75)	0.39
		II (2.20 ± 1.85)	III (2.41 ± 1.97)	0.47
		II (2.20 ± 1.85)	IV (2.42 ± 1.75)	0.79
		III (2.41 ± 1.97)	IV (2.42 ± 1.75)	0.99
*APOF*		I (4.34 ± 2.27)	II (3.36 ± 2.83)	5.80e–03
		I (4.34 ± 2.27)	III (2.51 ± 3.44)	2.00e–05
		I (4.34 ± 2.27)	IV (2.61 ± 1.81)	0.1
		II (3.36 ± 2.83)	III (2.51 ± 3.44)	0.08
		II (3.36 ± 2.83)	IV (2.61 ± 1.81)	0.43
		III (2.51 ± 3.44)	IV (2.61 ± 1.81)	0.91

**FIGURE 10:**
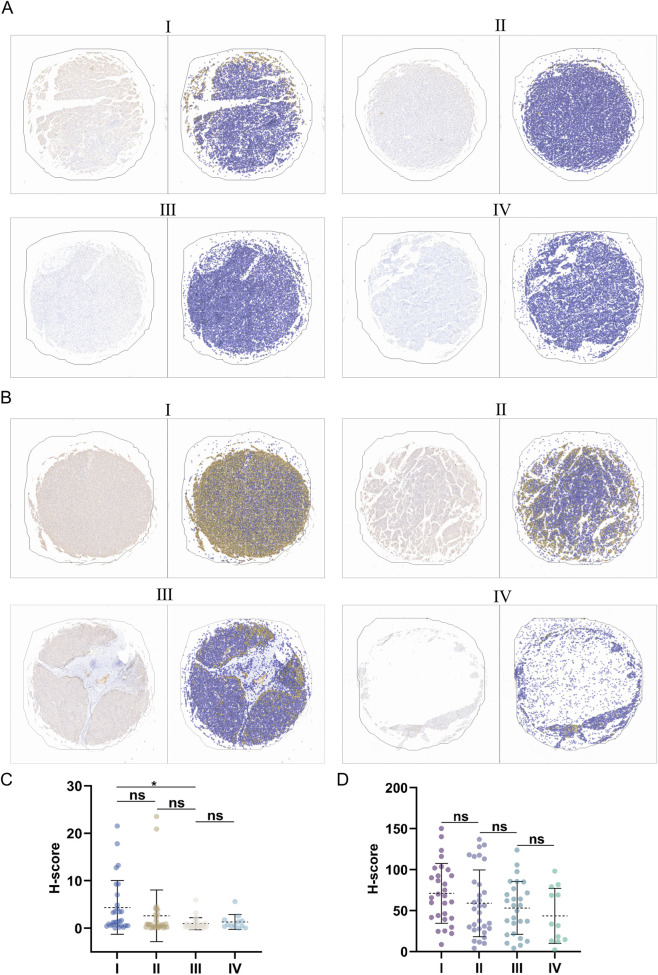
**(A,B)** IHC analysis of DNASE1L3 and APOF expression in LIHC patients across tumor stages. **(C,D)** H-Score of DNASE1L3 and APOF across LIHC clinical stages. ^*^ *p* < 0.05; ns, not significant.

Then, to verify the results of the bioinformatics analysis, we performed IHC staining on the tissue microarray slides. A significant decrease in DNASE1L3 protein levels was observed between stage I and stage III (*p* < 0.05, Figures 10A,C). APOF levels across all stages gradually decreased (*p* > 0.05, Figures 10B,D). Furthermore, an exploratory analysis comparing advanced stages (III and IV) against stage I is provided in Supplementary Figure S6. The results revealed a significant difference between the advanced stages and stage I (*p* < 0.01), further bolstering the conclusion that *DNASE1L3* expression is dysregulated in LIHC progression. This nonlinear pattern suggested a distinct biological role of *DNASE1L3* at different stages of LIHC progression.

### 3.6 *DNASE1L3* serves as an independent prognostic factor for LIHC patient survival

**FIGURE 11:**
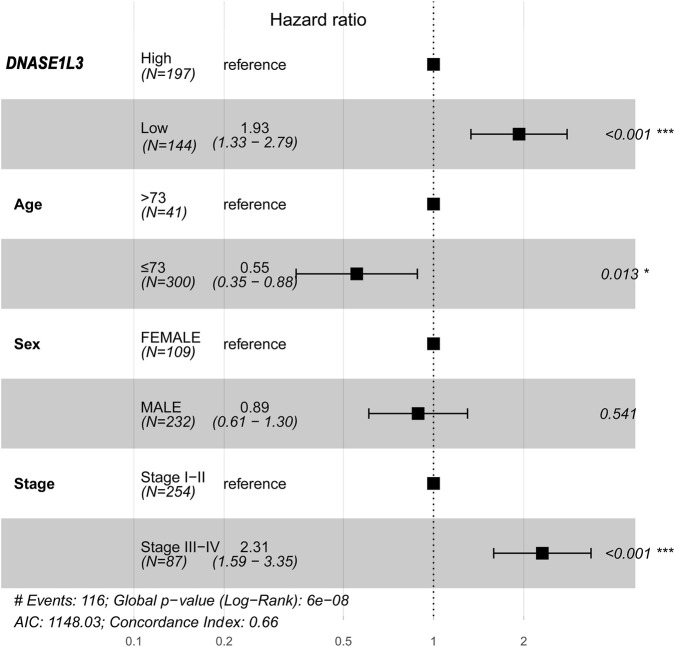
Forest plot of the multivariable Cox regression analysis for overall survival. ^*^ *p* < 0.05; ^***^ *p* < 0.001.

To evaluate whether *DNASE1L3* expression holds independent prognostic value beyond established clinical parameters, we performed multivariable Cox proportional hazards regression analyses for overall survival (OS). In the model adjusting for key covariates (age, sex, and clinical stage), low *DNASE1L3* expression remained a significant and independent risk factor for worse OS (HR = 1.93, 95% CI: 1.33–2.79, *p* < 0.001, Figure 11). These results demonstrate that the prognostic power of *DNASE1L3* is not confounded by other clinical features and that it provides complementary predictive information for LIHC patient outcomes.

## 4 Discussion

LIHC is a leading cause of cancer-related mortality worldwide, fueled by viral hepatitis, metabolic disorders and immune dysregulation (Rumgay et al., 2022; Ma et al., 2020; Zhang et al., 2017; Kramer et al., 2022; Wang et al., 2020a; Singh et al., 2021; Liu et al., 2022). Although transarterial chemoembolization and systemic agents such as sorafenib are the standard of care, their efficacy is modest, and cancer stem cells and tumor relapse further limit positive outcomes (Nio et al., 2017). Nanomedicines, exosome-based therapeutics, and magnetic hyperthermia are emerging treatment modalities that show promise for overcoming these hurdles (Shao et al., 2016; Huang et al., 2024; Zelli et al., 2022). Compounding these challenges, most patients are diagnosed at an advanced stage, resulting in a 5-year survival rate for LIHC of less than 20% (Li et al., 2020). To better understand the molecular drivers of this progression, we investigated *DNASE1L3*, a gene with putative tumor-suppressive functions. Our work uncovered a progressive loss of *DNASE1L3* from stage I to stage III in LIHC tumor tissue. Multivariable Cox regression analysis identified low *DNASE1L3* expression as an independent predictor of adverse clinical outcomes in LIHC. This stage-dependent downregulation suggested that *DNASE1L3* may be both a prognostic marker and a potential tissue-based biomarker for LIHC staging.

Cell cycle, DNA replication, and base excision repair signaling pathways were significantly upregulated in LIHC tumor tissue, while arachidonic acid metabolism, retinol metabolism, and tryptophan metabolism pathways were significantly downregulated. Differential gene expression analysis was performed on the three validation datasets, which identified 7 upregulated genes and 18 downregulated genes. Combining these genes with the core genes from the purple module, three common genes were identified: *DNASE1L3*, *APOF*, and *FCN3*. Using the UCSC database, we further investigated the functions of these three genes and found that *DNASE1L3* and *APOF* were closely associated with LIHC prognosis. An analysis using the MEXPRESS database confirmed a significant correlation between these two genes and LIHC clinical stage. To validate their clinical applicability in LIHC staging, we used the SangerBox gene and clinical staging module to analyze the genes in different clinical stages. The analysis revealed significant differences in *DNASE1L3* and *APOF* expression from stage Ⅰ to stages II and III, suggesting their potential as biomarkers for LIHC clinical staging.

Serum levels of DNASE1L3 were shown to be significantly lower in patients with LIHC than in healthy controls (Xiao et al., 2022). *In vitro* studies showed that DNASE1L3 inhibited cell proliferation by inducing G0/G1 phase arrest and apoptosis in LIHC cells (Xiao et al., 2022). In addition, the study results of Bo Li et al. showed that DNASE1L3 promoted the ubiquitination and degradation of β-catenin and inhibited epithelial-mesenchymal transition signaling, thus effectively inhibiting the development of LIHC (Li et al., 2022). Furthermore, LIHC patients with high expression of *DNASE1L3* were found to have a better prognosis (Wang et al., 2020b; Guo et al., 2021). Our immunohistochemical validation revealed that APOF levels remained unchanged. Pairwise comparisons revealed a complex dynamic of DNASE1L3. The most consistent finding was the significant downregulation of DNASE1L3 in stage III tumors compared to stage I. The fact that significance was shown between stage I and stage III, rather than between adjacent stages, suggested that the change in DNASE1L3 levels is a gradual process. In the clinical context of advanced LIHC (Liu et al., 2024), a combined exploratory analysis also showed significantly lower levels in stages III and IV than in stage I, supporting the concept that *DNASE1L3* loss of expression is a feature of progressive disease. However, the distinct increase in expression in stage IV warrants further investigation as it may indicate a context-dependent role for this protein in terminal-stage biology. To further investigate its clinical relevance, a multivariable Cox model was employed to assess the association between *DNASE1L3* expression and patient survival. The analysis confirmed that low *DNASE1L3* expression is an independent predictor of poor survival, supporting its potential as a prognostic biomarker in LIHC.

The Barcelona Clinic Liver Cancer (BCLC) and tumor–node–metastasis (TNM) systems are critical for initial treatment stratification based on tumor burden, liver function and performance status (European Association for the Study of the Liver, 2025). However, the heterogeneity in patient outcomes within the same stage remains a significant clinical challenge (Tian et al., 2019; Borde et al., 2022). Including a *DNASE1L3* assessment could help identify a high-risk subset of patients with early-stage disease who are otherwise predicted to have a favorable prognosis. Furthermore, *DNASE1L3* expression may contribute to a more refined prognostic stratification to aid in selecting between locoregional and systemic therapies. Prospective studies are warranted to validate the utility of *DNASE1L3* as a useful adjunct to the BCLC or TNM systems.

Our study has certain limitations that warrant consideration. The most significant limitation is the relatively small sample size in certain clinical stages, particularly stage IV, within our tissue microarray cohort. This imbalance inherently reduced the statistical power of pairwise comparisons involving this group and increased the uncertainty around the estimated effect sizes. Moreover, the nonsignificant trends in DNASE1L3 levels observed between some adjacent stages (e.g., I vs. II, II vs. III) may have reached statistical significance with a larger sample size, potentially further refining the progressive nature of the initial decline. Nonetheless, we posit that the statistically significant difference between stage I and stage III, which constitutes the core of the “descending phase,” remains robust. Future multicenter studies with extensive tissue sample collection are needed to definitively confirm the dynamic role of *DNASE1L3* throughout LIHC staging.

## 5 Conclusion

Our findings demonstrated a significant association between *DNASE1L3* expression and the clinical staging of LIHC patients, indicating that *DNASE1L3* may critically affect LIHC progression. This novel insight enhances our understanding of the pathophysiology of LIHC and offers potential avenues for the development of new diagnostic or prognostic biomarkers for LIHC.

## Data Availability

The datasets presented in this study can be found in online repositories. The names of the repository/repositories and accession number(s) can be found in the article/Supplementary Material.

## Abbreviations

CI, confidence intervals; GO, Gene Ontology; GS, gene significance; GSEA, gene set enrichment analysis; HR, hazard ratios; IHC, immunohistochemistry; KEGG, Kyoto Encyclopedia of Genes and Genomes; LIHC, liver hepatocellular carcinoma; MM, module membership; NAFLD, nonalcoholic fatty liver disease; TCGA, The Cancer Genome Atlas; TOM, topological overlap matrix; WGCNA, weighted correlation network analysis.
