# Supplementary material for: Identification of DNASE1L3 as a novel biomarker of clinical stage in liver hepatocellular carcinoma
Source: Front Mol Biosci. 2026 Jan 12;12:1681888. doi: 10.3389/fmolb.2025.1681888 (PMC12832532; doi:10.3389/fmolb.2025.1681888)
Supplement: Supplementary file 2 [file Image1.pdf]

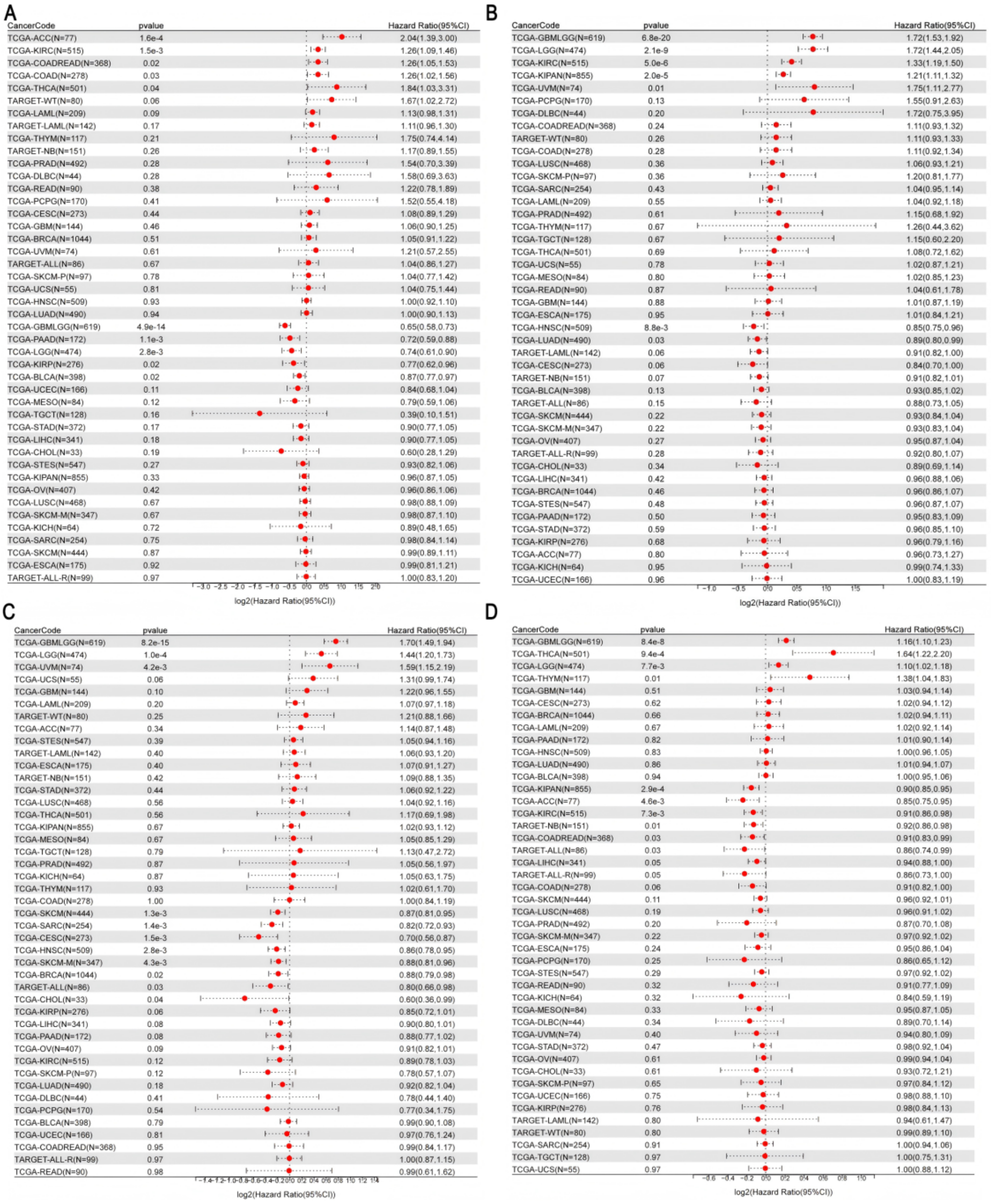

Figure S1. (A–D) Relationship between gene expression (*ADAMTS13*, *ANGPTL6*, *CFP*, and *COLEC10*) and prognosis of LIHC patients.

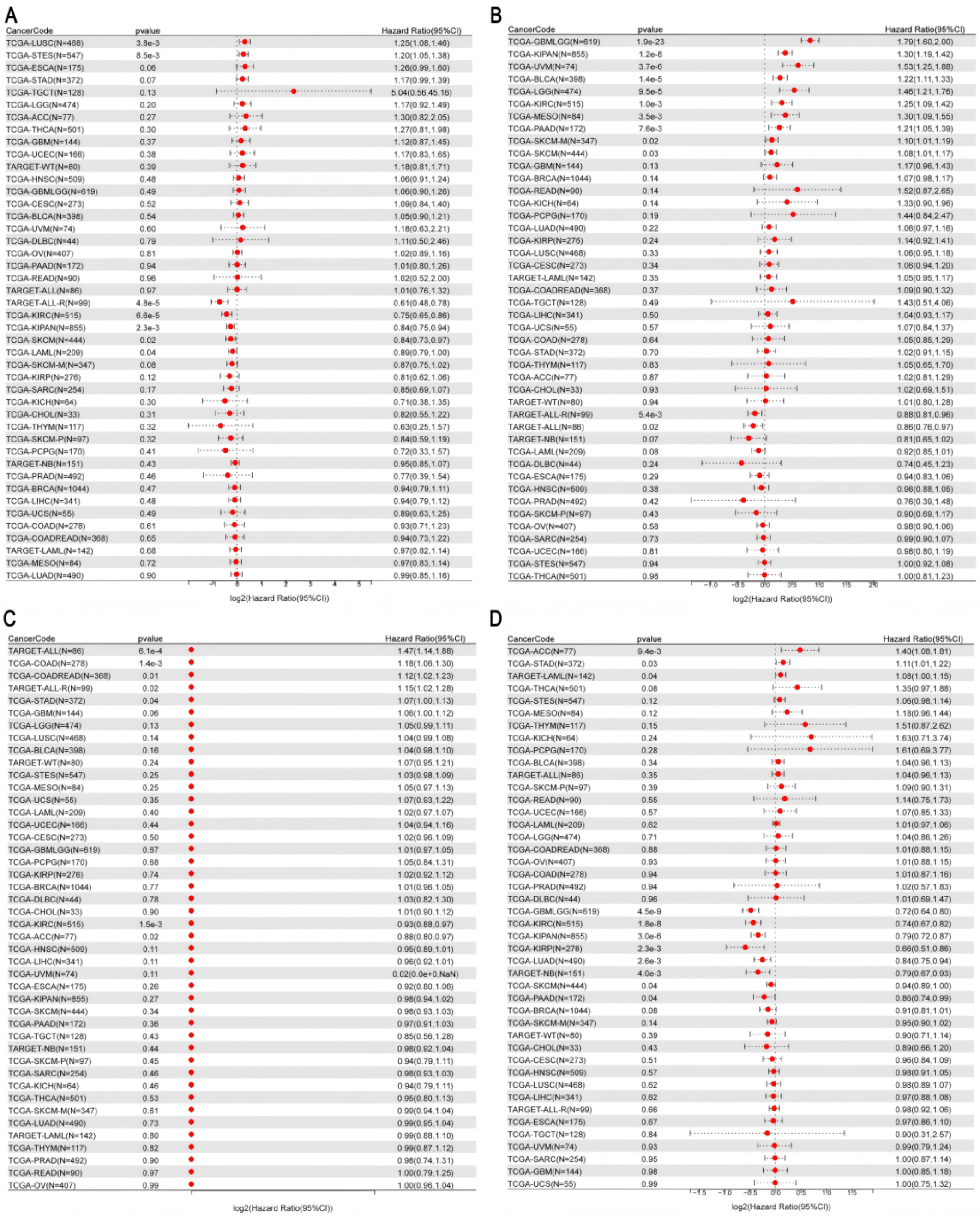

Figure S2. (A–D) Relationship between gene expression (*CSRNP1*, *ECM1*, *FCN2*, and *LIFR*) and prognosis of LIHC patients.

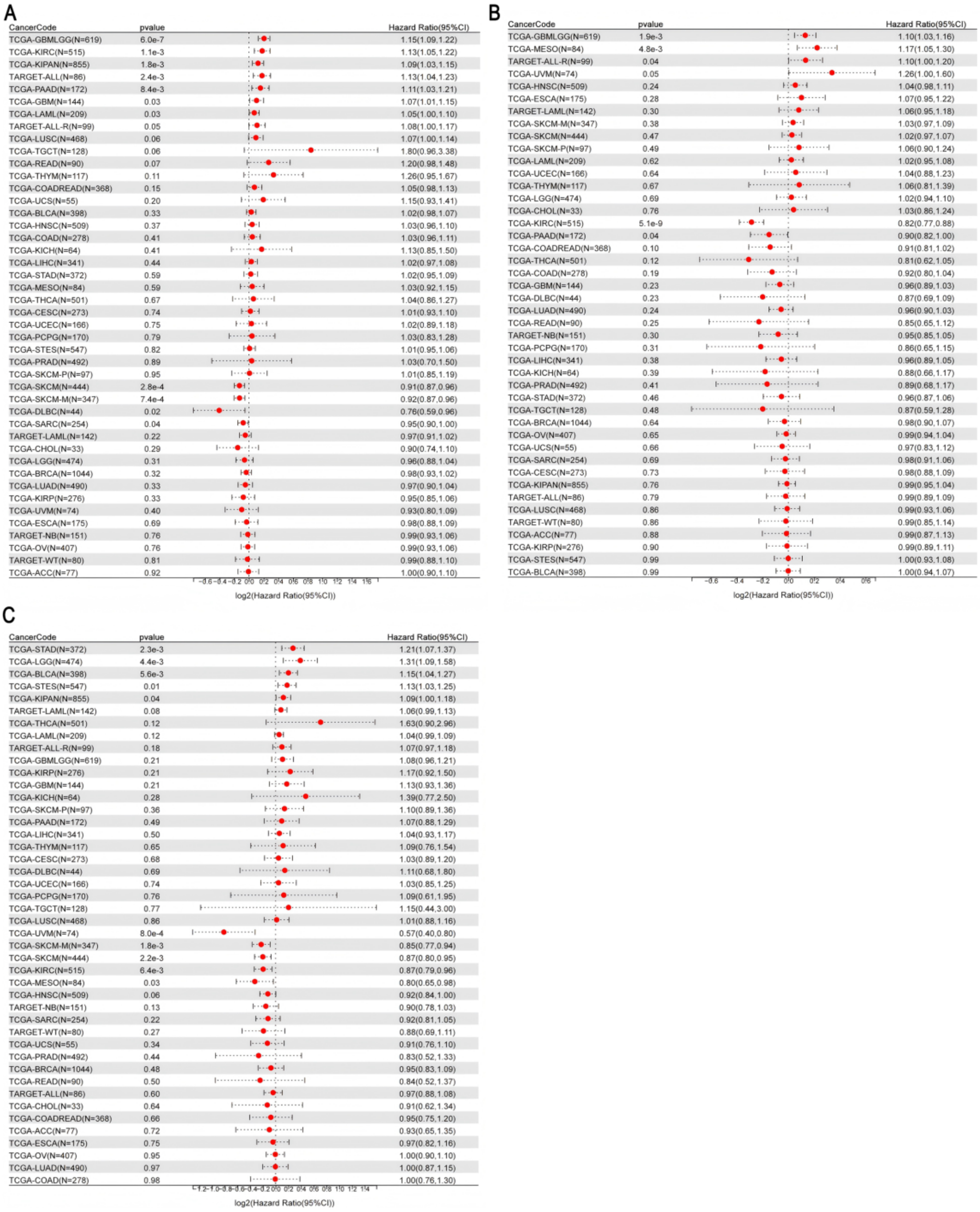

Figure S3. (A–C) Relationship between gene expression (*MARCO*, *OIT3*, and *PLSCR4*) and prognosis of LIHC patients.

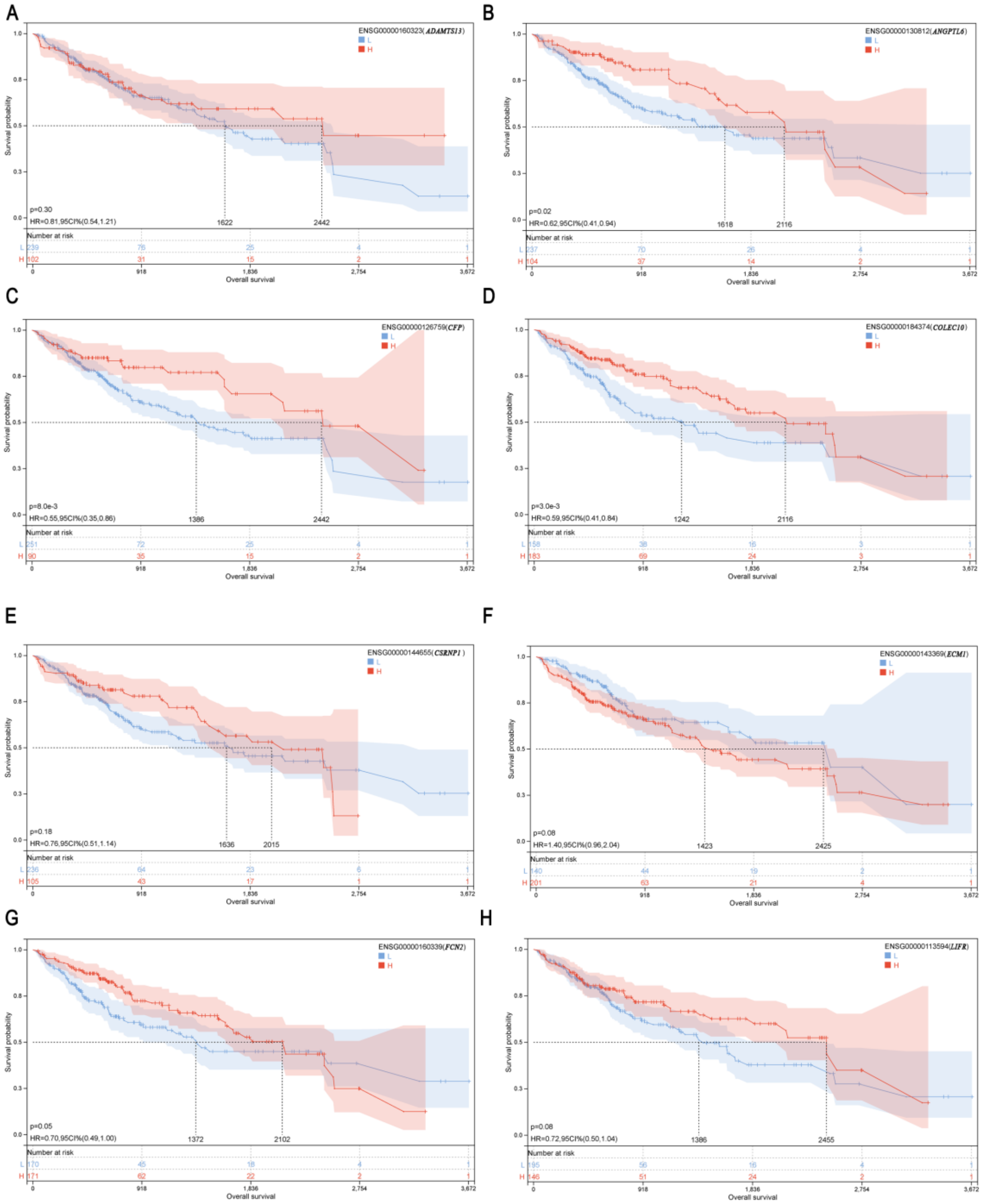

Figure S4. (A–H) Kaplan–Meier curves depicting patient survival based on gene expression (*ADAMTS13*, *ANGPTL6*, *CFP*, *COLEC10*, *CSRNP1*, *ECM1*, *FCN2*, and *LIFR*).

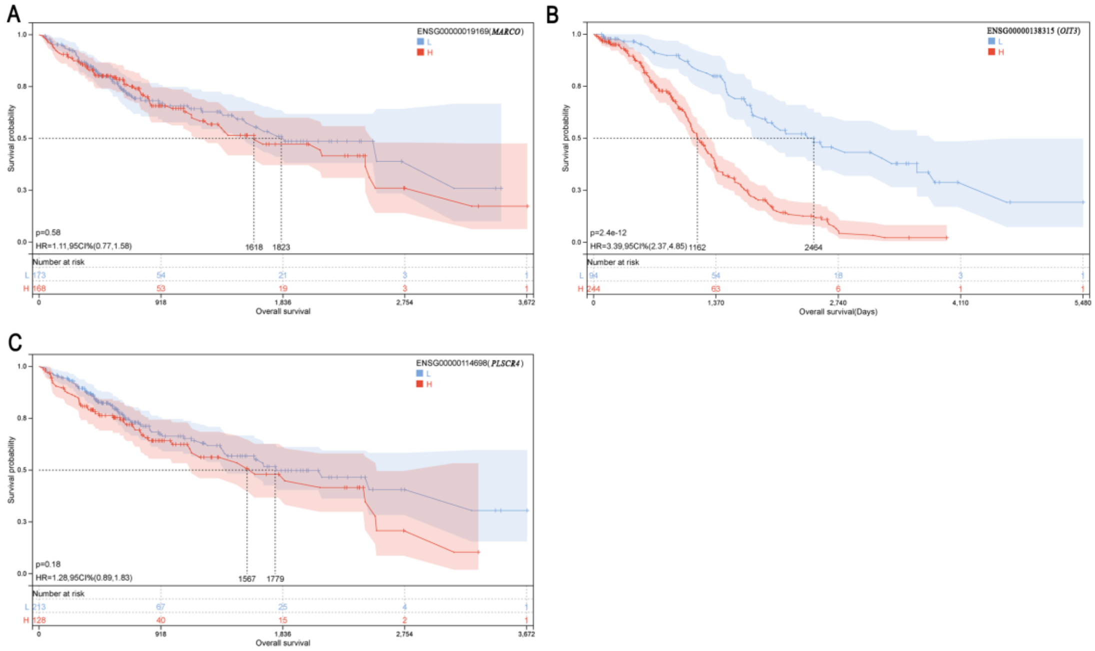

Figure S5. (A–C) Kaplan–Meier curves depicting patient survival based on gene expression (*MARCO*, *OIT3*, and *PLSCR4*).

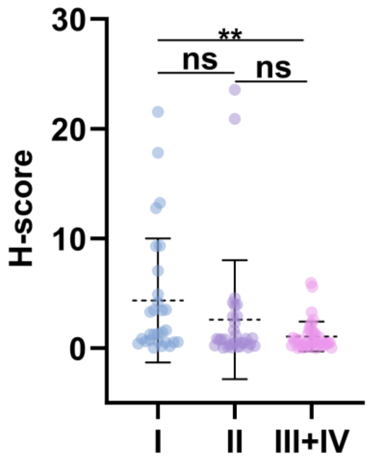

Figure S6. H-Score of DNASE1L3 in stage I, stage II, and advanced stages (III+IV).

****p < 0.01; ns, not significant.**
